# Acute Keratoconus-Like Hydrops after Laser In Situ Keratomileusis

**DOI:** 10.1155/2009/363482

**Published:** 2010-03-08

**Authors:** Carsten H. Meyer, Stefan Mennel, Jörg C. Schmidt

**Affiliations:** Department of Ophthalmology, University of Bonn, Ernst-Abbe Street 2, 53127 Bonn, Germany

## Abstract

*Purpose.* To demonstrate keratoconus-like hydrops after laser in situ keratomileusis (LASIK) by optical coherence tomography (OCT). *Patient and Methods.* A 21-year-old man received uneventful bilateral LASIK. On slit lamp examination or corneal topography there were no signs of fruste keratoconus. The preoperative corneal thickness was 587-*μ*m OD and the calculated ablation for two treatments was 114-*μ*m. Uneventful LASIK with an optical zone of 7 mm and an ablation of 89-*μ*m OD, and an ablation of 73-*μ*m OS was performed. Three years postoperatively, he complained about progressive myopia and impaired vision OD. His VA was hand motion OD and 20/20 OS. *Results.* OCT and light microscopy revealed an anterior corneal steepening and acute keratoconus-like excessive edematous swelling. *Conclusion.* The cornea is mechanically weakened after LASIK by the lamellar cut and tissue subtraction. Although the advocated minimal residual stromal bed thickness is 250-*μ*m, it may not be adequate to prevent progressive keratoconus-like hydrops in the selected cases.

## 1. Introduction

Acute keratoconus-like corneal swelling is associated with anterior corneal steepening. After laser in situ keratomileusis (LASIK) the integrity of the posterior stromal bed is excessively compromised by the disruption of corneal lamellae that occurs when the microceratom passes through the stroma and a consecutive excimer laser photoablation is performed [1]. This substantial reduction of the corneal biomechanical effective stress-bearing thickness may induce lateral pulling of the peripheral cornea and flattening of the central cornea [2]. There is concern that, at some point, the tensile strength of the cornea might be reduced to the degree that progressive ectasia ensues, thus resulting in corneal steepening, progressive myopia and irregular astigmatism. It has been stated that a residual stromal thickness of at least 250-*μ*m is necessary to forestall iatrogenic keratectasia after LASIK [2]. We present the tomographic features of a patient who developed progressive corneal hydrops after LASIK after a moderate ablation below 120-*μ*m.

## 2. Case Report

A 21-year-old man, required refractive surgery to correct his myopia and astigmatism at another hospital. His manifest best-corrected visual acuity (VA) was 20/20 with −1.75–1.75/90 OD and 20/20 with −3.0 sph OS. Preoperative corneal pachymetry measurements were 587-*μ*m OD and 594-*μ*m OS. Corneal topography was normal with no signs of frust keratoconus. Anterior slit lamp examination prior to surgery revealed no characteristic corneal findings of keratoconus including Vogt's striae or Fleischer ring. The patient underwent bilateral full-correcting LASIK within an optical zone of 7 mm with 89-*μ*m OD and 73-*μ*m OS by one of the most experienced refractive surgeons in Germany. Three months postoperative the refraction was −2.0/180 OD and +0.25–0.75/120 with a best-corrected VA 20/20OU. A retreatment of the astigmatism required an additional ablation of 25-*μ*m OD. 

**Figure 1:**
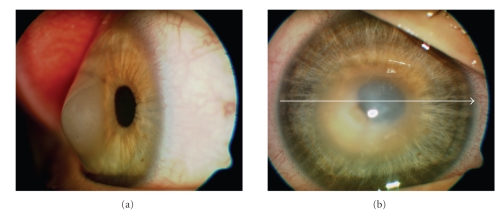
The photograph demonstrates the central keratectasia with an anterior corneal steepening from the side (1a). The thinned central cornea within the 7 mm central optical zone is “bulged” forward with a edematous swelling of approximately 1450 *μ*m similar to an acute keratoconus. Figure 1(b): the photograph of the frontside demonstrated the location of the corresponding OCT-scan.

**Figure 2:**
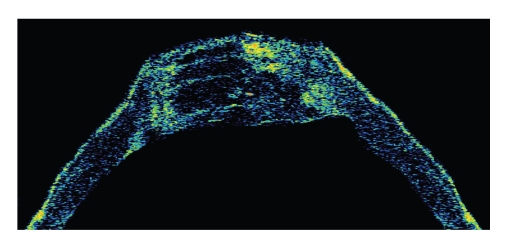
Horizontal OCT-scan displays a logarithmic false-colors image of the entire cornea OD composed of 7 images. Each OCT-scan has length of 4.07 mm represented by 100 columns and a depth of 1500 *μ*m represented by 300 pixels. OCT determined the cornea as a hyporeflective band in light greenish colors. While the lateral cornea has a normal thickness and appears steepend, the central cornea in a diameter of approximately 7.5 mm has a flatt curvature with an intrastromal low reflectivity, consistent with a severe intrastromal edema of approximately 1250 *μ*m.

**Figure 3:**
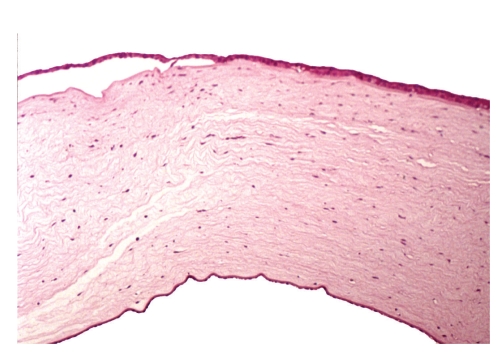
Light microscopy revealed a partially detached epithelium. The stromal architecture was rarified and the space between separate corneal lamellae edematous swollen. The descement and endothelial appeared undamaged.

 The patient came to our hospital seeking for a second opinion as he complained 6 months before the visit about diplopia and progressive impaired vision. Two days before the visit, his VA decreased to counting fingers OD and while his left eye remained stabile at 20/20. The diagnosis of acute keratoconus-like hydrops was made based on the progressive central edematous steepening of the corneal stroma (Figure 1) and a penetrating keratoplasty was recommended. Tomographic scans using optical coherent tomography (OCT) confirmed an anterior corneal steepening and acute keratoconus-like excessive edematous swelling of approximately 1250-*μ*m (Figure 2). The patient underwent an uncomplicated corneal transplantation (9.0 mm donor size to 8.5 mm recipient size) and the corneal tissue was sent for histologic evaluation. On light microscopy the stromal architecture appeared rarified and the space between separate corneal lamellae was edematously swollen (Figure 3).

## 3. Comment

We describe the unique tomographic features by OCT and histology in an keratokonus-like hydrops after LASIK. OCT may be promising in providing detailed analysis of the prior structural stromal architecture [3], during [4, 5] and after LASIK [6]. Similar to the OCT, the optical low coherent pachymetry (OLCR) can investigate the mean flap thickness after LASIK [7]. These novel techniques may help to determine the tomographic corneal architecture and anterior segment on a micrometer scale generating a valuable insight of the in vivo structure [8]. 

Our patient provides an instructive report of the risk of keratoconus-like hydrops following the LASIK of his moderate myopia and irregular astigmatism in the eyes. The preoperatively measured corneal thickness was 587-*μ*m OD and the calculated ablation for both treatments was 114-*μ*m, indicating a remaining thickness of approximately 470-*μ*m. 

 After LASIK, the cornea is mechanically weakened by the lamellar cut and the substantial tissue subtraction. Although the advocated minimal residual stromal-bed thickness is 250-*μ*m, it may not be adequate to prevent progressive keratoconus-like hydrops in the selected cases.
